# Editorial: Machine learning and deep learning applications in pathogenic microbiome research

**DOI:** 10.3389/fcimb.2024.1429197

**Published:** 2024-06-20

**Authors:** An-Tian Chen, Xinyan Wu, Gang Ye, Wenle Li

**Affiliations:** ^1^ Department of Cardiology, State Key Laboratory of Cardiovascular Disease, Fuwai Hospital, Chinese Academy of Medical Sciences & Peking Union Medical College/National Center for Cardiovascular Diseases, Beijing, China; ^2^ Department of Computer Science, College of Natural Sciences, The University of Texas at Austin, Austin, TX, United States; ^3^ College of Veterinary Medicine, Sichuan Agricultural University, Chengdu, China; ^4^ State Key Laboratory of Molecular Vaccinology and Molecular Diagnostics & Center for Molecular Imaging and Translational Medicine, School of Public Health, Xiamen University, Xiamen, China

**Keywords:** machine learning, application, predictive model, pathogenic microbiome, gut microbiota

Artificial intelligence has established a solid basis for deep learning (DL), especially with the introduction of the Transformer architecture, which has gained much attention from researchers in multiple disciplines. Machine learning (ML) and DL, branches of artificial intelligence, have increasingly transformed research in various fields. One area notably impacted is microbiology (Obermeyer and Emanuel, 2016). In particular, the complexity and diversity of microbiomes and infectious diseases make them ideal candidates for novel ML and DL techniques.

In this Research Topic, titled “*Machine Learning and Deep Learning Applications in Pathogenic Microbiome Research*”, we have gathered a collection of 11 manuscripts that embody the application of ML and DL in the research field of pathogenic microbiomes. These collected manuscripts are mostly original articles that provide an understanding of how ML and DL can be used to further insights into research on pathogenic microbiomes. Currently, ML is widely used in the development of predictive models (Collins and Moon, 2019). By integrating ML or DL approaches with predictive models, the manuscripts in this Research Topic highlight the importance of interdisciplinary integration in understanding diseases associated with pathogenic microbiomes and promoting better health and the well-being of both humans and ecosystems.

In this Research Topic, Shao et al. dive into the complex interactions between pathogenic microorganisms and various orthopedic conditions in the mini-review “*Exploring the impact of pathogenic microbiome in orthopedic diseases: machine learning and deep learning approaches*”. By analyzing datasets on microbiota and the interactions with the host, they highlight how ML and DL can enhance the understanding, diagnosis, and treatment of diseases such as osteoporosis and arthritis.

Gut microbiota impact human health and disease (Lynch and Pedersen, 2016). By utilizing ML and DL, relationships between microbiota and diseases can be established and understood, enhancing overall health and contributing to the management of diseases. In their manuscript entitled “*Gut microbiota landscape and potential biomarker identification in female patients with systemic lupus erythematosus using machine learning*”, Song et al. explore the relationship between gut microbiota and the autoimmune disease systemic lupus erythematosus (SLE) and aim to identify biomarkers for SLE by analyzing the gut microbiota in female patients using machine learning techniques. The article “*Gut microbiome-based noninvasive diagnostic model to predict acute coronary syndromes*” presents a study by Wang et al. on the potential of using a gut microbiome profile as a noninvasive diagnostic tool for acute coronary syndromes. “*Construction and validation of a machine learning model for the diagnosis of juvenile idiopathic arthritis based on fecal microbiota*”, by Tu et al., discusses the use of fecal microbiome profiling in developing a diagnostic tool for juvenile idiopathic arthritis, leading to development of the XGBoost model.

Chronic diseases are also closely linked to gut microbiota. The article by Wang et al., “*The effect and mechanism of Fushen Granule on gut microbiome in the prevention and treatment of chronic renal failure*”, examines the impact of Fushen Granule, a Traditional Chinese Medicine (TCM), on the gut microbiome and its therapeutic effects on chronic renal failure. For further study concerning TCM, Zeng et al. discuss the impacts of two Chinese herbal formulas on insomnia treatment via gut microbiome modulation in their research paper “*Traditional Chinese herbal formulas modulate gut microbiome and improve insomnia in patients with distinct syndrome types: insights from an interventional clinical study*”, enhancing our understanding of the gut-brain axis and supporting strategies for using TCM for insomnia. The study, “*Alteration and clinical potential in gut microbiota in patients with cerebral small vessel Disease*”, by Shi et al. examines changes in the gut microbiota of patients with cerebral small vessel disease and the potential clinical implications.

Apart from gut microbiota, microbiomes located in other parts of the body can also impact health. “*The impact of Sangju Qingjie Decoction on the pulmonary microbiota in the prevention and treatment of chronic obstructive pulmonary disease*” by Liu et al. examines the effects of Sangju Qingjje Decoction (SJQJD) on the pulmonary microbiota of COPD rats, finding that SJQJD improves lung structure, reduces inflammation, and enhances the diversity and abundance of lung microbiota. The study by Zheng et al. titled “*Comparative characterization of supragingival plaque microbiomes in malocclusion adult female patients undergoing orthodontic treatment with removable aligners or fixed appliances: a descriptive cross-sectional study*” investigates the effects of different orthodontic appliances on the oral microbiome. The study found that fixed appliances are associated with increased anaerobic and Gram-negative bacteria compared to clear aligners and could lead to the development of better cleaning techniques and materials. The authors emphasized the need for tailored oral hygiene practices for individuals undergoing orthodontic treatment.

In addition to studies related to human health, the collection of papers in this Research Topic includes content on animal experiments and single-cell analysis. In the original article, “*Comprehensive assessment of HF-rTMS treatment mechanism for post-stroke dysphagia in rats by integration of fecal metabolomics and 16S rRNA sequencing*”, Zhao et al. explore the effects of high-frequency repetitive transcranial magnetic stimulation (HF-rTMS) on swallowing dysfunction in post-stroke rats and reveal that HF-rTMS can improve swallowing function and modify gut microbiota composition. The study titled “*Integrating machine learning algorithms and single-cell analysis to identify gut microbiota-related macrophage biomarkers in atherosclerotic plaques*” by Ke et al. focuses on identifying macrophage biomarkers linked to gut microbiota that are influential in the development of atherosclerotic plaques, combining machine learning techniques and single-cell analysis.

In conclusion, the Research Topic “*Machine Learning and Deep Learning Applications in Pathogenic Microbiome Research*” encompasses 11 valuable manuscripts that integrate ML and DL into innovative microbiome research. The Research Topic features interdisciplinary applications ranging from diagnostic models to interactions between microbiomes and various diseases, highlighting the significance of understanding and managing health through the view of microbiota. We are thankful to the editorial team and authors for their significant contributions which have been instrumental to the success of this Research Topic.

## Author contributions

A-TC: Writing – original draft. XW: Writing – review & editing. GY: Writing – review & editing. WL: Writing – review & editing.
